# CYTOPLASMIC-MEMBRANE EGFR PREDICTS EXPANDED RAS MUTATION STATUS IN COLORECTAL CARCINOMAS?

**DOI:** 10.1590/0102-672020210001e1574

**Published:** 2021-06-11

**Authors:** Thiago David Alves PINTO, Thaís David das Neves ALVES, Sebastião Alves PINTO, Enio Chaves OLIVEIRA

**Affiliations:** 1 Department of Pathology, Goiano Institute of Oncology and Hematology, Goiânia, GO, Brazil; 2 Department of Pathology, Faculty of Medicine, Federal University of Goiás, Goiânia, GO, Brazil; 3 Department of Surgery, Faculty of Medicine, Federal University of Goiás, GO, Goiás, Brazil

**Keywords:** Colorectal cancer, Ras genes, Mutation, Epidermal growth factor receptor, Câncer colorretal, Genes RAS, Mutação, Receptor de fator de crescimento epidérmico

## Abstract

**Background:**

Inhibitors of the epidermal growth factor (EGFR) represent an effective therapeutic option for patients with metastatic colorectal carcinoma, free of activating mutations in KRAS and NRAS. However, the research of mutations is of high cost and scarcely accessible. The expression of the EGFR by immunohistochemistry predicting the mutation status of the expanded RAS (KRAS and NRAS), may allow treatment by a diagnostic method less costly and more accessible.

**Aim:**

Investigate the correlation between the clinical-pathological data, the cytoplasmic-membrane expression of the EGFR and the mutational status of the expanded RAS.

**Method:**

A total of 139 patients with colorectal carcinoma from the archives of Instituto Goiano de Oncologia e Hematologia were evaluated.

**Results:**

Mutation of the expanded RAS was detected in 78 (56.1%) cases. The EGFR expression was stratified in 23 (16.5%) “positive”, 49 (35.2%) "negative" and 67 (48.2%) "uncertain". No significant correlation was found between the mutational status of the RAS and the EGFR expression in comparison to age, gender, location, histological type, histological grade and stage. From 23 "positive” cases, 21 (91.3%) showed wild-type RAS gene, and 49 "negative”, 41 (83.7%) presented mutation, resulting in a strong association between EGFR "positive", "negative” groups and the mutational status of the RAS (p<0.001), with 86.1% of accuracy.

**Conclusions:**

The cytoplasmic-membrane analysis of the EGFR expression stratified into "positive", "negative" and "uncertain" predicts mutational status of the RAS in 51.7% of the cases (p<0.001), with 86.1% of accuracy.

## Graphical abstract

[Fig f1a]

**Figure f1a:**
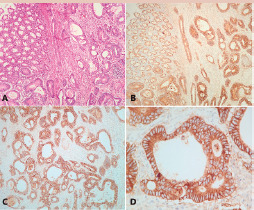
Colorectal carcinoma: H&E (A) and immunohistochemistry for EGFR (B-D)

[Fig f1as]

**Figure f1as:** Carcinoma colorretal: H&E (A) e imunoistoquímica para EGFR (B-D)

## RESUMO

**Racional:**

Inibidores do fator de crescimento epidermal (EGFR) representam opção de terapia efetiva para o câncer colorrectal metastático, na ausência de ativação de mutações KRAS e NRAS. Entretanto, a pesquisa de mutações é cara e pouco acessível. A expressão de EGFR por imuno-histoquímica predizendo o status mutacional do RAS expandido (KRAS e NRAS) poderia permitir o tratamento por método diagnóstico menos caro e mais acessível.

**Objetivo:**

Investigar a correlação entre os dados clinicopatológicos, a expressão de EGFR na membrana citoplasmática e o status mutacional do RAS expandido.

**Método:**

Estudo retrospectivo de acurácia envolvendo 139 pacientes com carcinoma colorretal.

**Resultado:**

A mutação do RAS expandido foi detectada em 78 (56,1%) casos. A expressão de EGFR foi estratificada em 23 (16,5%) casos “positivos”, 49 (35,2%) casos “negativos” e 67 (48,2%) “duvidosos”. Não houve correlação significante entre o status mutacional do RAS e a expressão de EGFR em relação a idade, gênero, local do tumor, tipo histológico, grau histológico e estádio clínico. Em 23 casos “positivos”, 21 (91,3%) mostraram gene RAS tipo selvagem, e em 49 “negativos”, 41 (83,7%) apresentaram mutação, resultando em forte associação entre grupos EGFR “positivo” ou “negativo” e o status mutacional do RAS (p<0.001), com 86,1% de acurácia.

**Conclusão:**

A análise da expressão de EGFR na membrana citoplasmática estratificada em “positivo”, “negativo” e “duvidoso” prediz o status mutacional do RAS em 51,7% dos casos (p<0.001), com 86,1% de acurácia.

## INTRODUCTION

The first drug with proven action to treat advanced colorectal cancer (CRC) was the 5-fluorouracil. Posteriorly, the association with leucovorin improved the outcomes. Some years ago, two new drugs were added to the treatment protocols: irinotecan and oxaliplatin. Recently two new classes of biological agents were developed to treat colorectal cancer targeting the Vascular Endothelium Growth Factor (VEGF) and Epidermal Growth Factor Receptor (EGFR)^18^. Many factors associated to survival (prognostic biomarkers) and factors able to identify patients with low or higher probability to get a benefit from particular treatment (predictive biomarkers) need a better understanding. Those biomarkers are auxiliary tools to select appropriated patients to the right chemotherapy protocol using association with anti-VEGF or anti-EGFR^5^. 

Monoclonal antibodies inhibitors of the epidermal growth factor receptor (iEGFR) represent a therapeutic option with proven efficacy for patients with metastatic CRC and wild-type KRAS and NRAS genes. When there are mutations of these genes there is a constitutive activation of the transduction signs from them and the tumor cells become insensitive to the iEGFR^5^ ^,^ ^18^. 

It has been proposed the test by PCR and gene sequencing to detect activating mutations at codons 12 and 13 (exon 2), 59 and 61 (exon 3), 117 and 146 (exon 4) of the KRAS and NRAS genes before the start of therapy, due to the inhibitors being high cost drugs and with deleterious effects in patients with mutation^3^. However, the mutation research by PCR and gene sequencing is of high cost, time-consuming, being performed only in large centers^18^.

The EGFR expression by immunohistochemistry has already been investigated to guide the treatment of these patients, but there was disagreement between the expression and the therapeutic response in previous studies^22^. In these studies, it was used the antibody anti-EGFR clone 2-18C9 PharmDX^TM 2,24^ and considered as positivity only the membrane expression. However, the expression and activation of the receptor occur through early and late regulatory loops, that involve the RAS, attenuating the signal across the whole cascade and/or internalizing the receptor, possibly resulting in dephosphorylation or degradation, providing different degrees of cytoplasmic and membrane marking^2^. 

Mutated and constitutively activated KRAS and NRAS may activate the regulatory loops, leading to the internalization of the EGFR and its cytoplasmic marking, interfering in their expression pattern^6^ (Figure 2). In addition, the use of a monoclonal anti-EGFR wild-type antibody (DAK-H1-WT) in CCR has presented significant gain in sensitivity in relation to the antibody used in other studies (EGFR 2-18C9 PharmDX^TM^)^2^.

Therefore, the validation of the cytoplasmic-membrane expression of the EGFR by immunohistochemistry, predicting the mutational status of the expanded RAS, may allow that the treatment to be established through a diagnostic method less costly and more accessible.

The objective of this study was to propose an analysis method of the EGFR expression, using the monoclonal anti-EGFR DAK-H1-WT antibody, considering membrane/cytoplasmic marking and evaluate its correlation with the mutational status of the expanded RAS.

## METHODS

This study was approved by Research Ethics Committee from Alberto Rassi Hospital under ID 961.174 - CEPHGG 775/150.

### Patient selection

The archive of the Department of Pathology of Instituto Goiano de Oncologia e Hematologia, Goiânia, GO, Brazil, was researched for the selection of patients who underwent surgical resection of CCR in the period from January 2014 to June 2015. A total of 169 cases with available tumor biological material paraffin block were selected. Thirty samples with signs of autolysis, unsuitable for molecular study or that had not been tested for mutation of the RAS were excluded.

Clinical-pathological data and the mutational status of the expanded RAS in each patient were obtained from our database. Type, histological grade and location were also analyzed in accordance with the criteria established by the World Health Organization in 2010 (http://www.tumourclassification.iarc.who.int) and the staging defined in accordance with the 7^th^ edition of the staging manual of the American Joint Committee on Cancer (http://www.cancerstating.org).

The sample size was calculated based on the population proportion estimate, considering 0.05 of confidence level, test power of 90% and that patients with RAS mutations represent 45% of the bearer population of CCR.

### PCR reactions and gene sequencing

All the selected cases were tested, without cost for the study or for the patients, on clinic demand, for the mutation of the expanded RAS by “GENteorienta” (Merck, Darmstadt, Germany) and RASTREAR (AMGEN, Thousand Oaks, USA) programs, in the certificated associated laboratory Progenética (Rio de Janeiro, Brazil). For DNA extraction from paraffin blocks, areas containing at least 50% of tumor cells were delimited, by a pathologist, in stained slide with H&E, for each case. The non-stained slides, corresponding, were immersed in xylol (Sigma, St Louis, USA) and twice in alcohol 100% (Merck, Darmstadt, Germany), for 5 min each. Tumor areas, which were previously delimited by comparison with the corresponding slides of H&E, went through microdissection and were transferred to a tube of microcentrifuge. The DNA was isolated using QIAamp^®^ DNA FFPE Tissue Kit (Qiagen, Hilden, Germany), following the manufacturer instruction. Finally, the DNA was quantified by spectrometry with NanoDrop-1000^®^ (NanoDrop Technologies, ThermoFisher, Waltham, USA^®^).

All samples were subjected to DNA sequencing. The exons 2 (codons 12 and 13) and 3 (codon 61) of the KRAS were tested by pyrosequencing with the KRAS Pyro Kit (QIAGEN) commercial kit. The reactions and analysis were performed according to the manufacturer’s instructions, using the PyroMark Q24 system (QIAGEN). 

The exon 4 of the KRAS and all exons of the NRAS have been tested by the automated Sanger method in a two-sided tape (forward and reverse) in order to evaluate the existence of DNA changes in both the tapes. The primer pairs in this study for NRAS exons 2, 3 and 4, and KRAS exon 4 were all designed using the software primer-BLAST (http://www.ncbi.nlm.nih.gov/tools/primer-blast).

In the analysis of all exons the DNA was subjected to initial denaturation, followed by 35-40 cycles of amplification. 

Before the sequencing all amplification products were purified to remove primer, salts, enzymes and dNTP’s excesses of the prior reaction.

The sequencing reaction used 1µL of product of each sample added to 0.5 µL of Big Dye^®^ Terminator v1.1 sequencing Ready Reaction Mix (Applied Biosystems, Foster City, USA), 3.4 µL of Big Dye^®^ Terminator v1.1, v1.3 5x sequencing buffer (Applied Biosystems), 350 nM of one of the primers (“forward” and “reverse”) and 4.78 µL of double-distilled sterile water (B. Braun, Melsungen, Germany), for a total volume of 10 µL. The samples were then subjected to an initial denaturation, followed by 35 cycles of synthesis. The sequencing products were purified using Illustra Sephadez^®^ G-50fine (GE Healthcare Life Sciences) and plus 12 µL of Hi-DiTM^®^ Formalize (Apllied Biosystems). The products were analyzed in the ABI PRISMTM 310^®^ Genetic Analyzer (Applied Biosystems) or in 3500^®^ Genetic Analyzer (Applied Biosystems). Electroferrograms were analyzed with the Sequencing Analysis Software v5.4 (Applied Biosystems). All of them have been read at least twice and reviewed manually and with the software Mutation Surveyor Software v4.0.8.

### Reactions of immunohistochemistry

The reactions for detection of the EGFR expression were performed in all the selected cases. Two slides of each FFPE sample with cuts of 4µ of thickness were obtained. One of the slides were stained with H&E, for confirmation of morphological data, and the other submitted the reaction of immunohistochemistry by automated Dako Link Autosteiner48^®^ method (Dako, Glostrup, Denmark), using Dako Flex kit. The samples were subjected to antigen retrieval by controlled heat exposure in a solution of high pH using PTLink^®^ (Dako), endogenous blockade with peroxide, incubation with the primary antibody Epidermal Growth Factor Receptor - Clone DAK-H1-WT - Anti-Human - Monoclonal Mouse (Dako) at a dilution of 1:1000, amplification with LINKER (EnVision FLEX - Dako), reaction with the polymer (EnVision Flex/HRP) for 20 min, revelation of chromogen for 5 min and counterstaining with hematoxylin (EnVision FLEX) for 5 min. 

### Analysis of the immunohistochemistry slides

The slides reaction of each case was evaluated by two pathologists in consensus. We used a classification score of the membrane and cytoplasm marking (Table 1). For membrane marking the score ranged from 0 to +3. The sample should contain strong and complete membrane marking in more than 50% of neoplastic cells to be considered +3 (Figure 2D). The focal and weak membrane marking in any quantity of cells was considered +1 (Figure 2A) and the rest of the cases were considered +2 (Figures 2B and 2C). The cytoplasmic marking ranged from 0 to -3, whereas since the absence of marking (0) until the strong and diffuse marking (-3, Figure 2). The cases with large negative areas, larger than 30% of the neoplasia, both membrane and cytoplasm were also considered, independently of the marking, assigning score of -1 to these cases (Table 1).

**TABLE 1:** Score proposed for analysis of the cytoplasmic-membrane expression of the EGFR by immunohistochemistry

Membrane (m)	Criteria
Score 3+	Strong and complete in more than 50% of the cells
Score 2+	Intermediate between score 1+ and 3+
Score 1+	Focal and weak in any quantity of cells
Score 0	Absence of membrane marking
Cytoplasm (c)	Criteria
Score 0	Absence of cytoplasmic marking
Score 1-	Weak in any quantity of cells
Score 2-	Intermediate in more than 10% of the cells
Score 3-	Strong in more than 10% of the cells
Negative areas (na)	Criteria
Score 0	Any marking in more than 70% of the cells
Score 1-	Absence in making in more than 30% of the cells
Result	Criteria
Positive	M + C + NA ≥2
Negative	M + C + NA ≤1-
Uncertain	M + C + NA =0 or 1.

**FIGURE 1:**
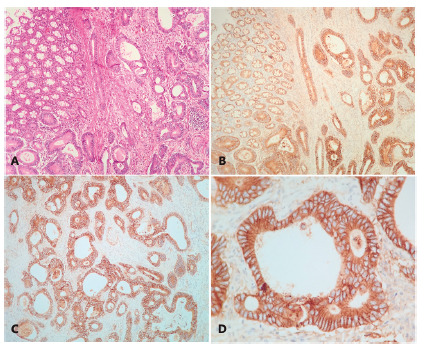
A) Grade II CCR with normal mucosa area on the upper left corner (H&E 40x); B) immunohistochemistry for EGFR showing strong marking of the membrane and weak of the cytoplasm (M3C1) Score 2 (positive) Wild-type RAS; C and D) details in other areas of the same case showing homogeneous pattern of the M3C1 marking

The cases were grouped according to scores sum into 3 classes. “positive”, “negative” and “uncertain”. The samples with results 2 and 3 were allocated in the “positive” class (Figure 1). Those with 0 and 1 results were assigned in the “uncertain” class. Those with negative results were assigned in the “negative” class.

**FIGURE 2:**
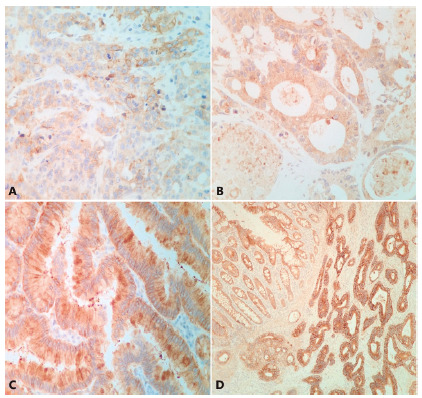
A) Immunohistochemistry for EGFR showing weak marking of membrane and cytoplasm (M1C1) score 0 (uncertain) wild-type RAS; B) negative membrane and cytoplasm 2+ (M0C2) score -2 (negative) mutated RAS KG12A; C) membrane +1 and cytoplasm +3 (M1C3) score -2 (negative) mutated RAS KG12V; D) membrane +3 and cytoplasm +1 (M3C1) score 2 (positive) wild-type RAS. On the upper left corner normal mucosa with tenuous cytoplasmic-membrane marking.

### Statistical analysis

The statistical analysis was performed using the software SPSS 18.0 (SPSS Inc., Chicago, IL, USA). To evaluate the statistical significance, it was applied the chi-square test considering p<0.001.

## RESULTS

### Clinical-pathological correlations of the mutation of the expanded RAS and of the EGFR expression

The average age of the 139 patients was 61.8 years with a standard deviation of 13.9 years, being 26 (19.5%) less than 50 years and 10 (7.5%) less than 40. There was no disparity of gender, being 52.9% of men and 47.1% of women (Table 2). 

**TABLE 2:** Clinical-pathological correlation between EGFR and RAS

	EGFR (n/%)				RAS (n/%)		
	Positive	Negative	Uncertain	p	Savage	Mutated	p
Age							
<40 years	1/10.0	2/20.0	7/70.0	0.65	5/50.0	5/50.0	0.54
40 to 50 years	1/6.3	6/37.6	9/56.3		5/31.36	11/68.8	
>=50 years	20/18.7	38/35.5	49/45.8		48/49.9	59/55.1	
Gender							
Male	15/20.5	23/31.5	35/47.9	0.54	35/47.9	38/52.1	0.34
Female	8/12.3	26/40.0	31/47.7		26/40.0	39/60.0	
Location							
Right Colon	7/14.6	15/31.3	26/54.2	0.09	18/37.5	62.5//30	0.39
Left Colon	5/17.9	9/32.2	14/50.0		15/53.6	13/46.4	
Recto-sigmoid	11/17.5	25/39.7	27/42.9		28/44.4	35/55.6	
Histological type							
Tubular	20/17.1	43/36.7	54/46.2	0.69	54/46.2	63/53.8	0.19
Mucinous	2/25.0	1/12.5	5/62.5		4/50.0	4/50.0	
Tubular-mucinous	1/7.1	5/35.7	8/57.1		3/21.4	11/78.6	
Histological degree							
1	1/14.3	2/28.6	6/24.0	0.18	3/42.9	4/57.1	0.90
2	16/15.0	41/38.3	6/24.0		46/43.0	61/57.0	
3	6/24.0	50/46.7	13/52.0		12/48.0	13/52.0	
Stage							
0	0/0.0	2/11.0	6/9.0	0.05	4/6.6	4/5.1	0.53
I	1/4.3	6/33.1	9/13.4		4/6.6	12/15.4	
IIA	8/34.8	14/54.7	16/23.9		16/26.2	22/28.2	
IIB	0/0.0	1/2.7	5/7.5		3/4.9	3/3.8	
IIC	3/13.0	1/2.7	0/0.0		3/4.9	1/1.3	
IIIA	0/0.0	3/8.1	1/1.5		3/4.9	1/1.3	
IIIB	5/21.7	15/40.5	22/32.8		18/29.5	24/30.8	
IIIC	5/21.7	7/18.9	8/11.9		9/14.8	11/14.1	
IV	1/4.3	0/0.0	0/0.0		1/1.6	0/0.0	

No significant correlation was found between the mutational status of the expanded RAS and the EGFR expression in comparison to age (p=0.541 and 0.652 respectively), gender (p=0.348 and 0.540), location (p=0.393 and 0.098), histological type (p=0.199 and 0.697), histological grade (p=0.900 and 0.182) and stage (p=0.533 and 0.053, Table 2).

The mutation of the expanded RAS was detected in 78 (56.1%) of the 139 cases with CCR tested by pyrosequency and by automatized Sanger method. Of these, 72 cases (51.79%) had mutations in the KRAS gene, with 63 cases (45.3%) with mutations in the codons 12 and 13 (exon 2), four cases (2.8%) with mutations in codon 61 (exon 3) and five cases (3.59%) with mutations in codon 146 (exon 4). No mutations were detected in codons 59 (exon 3) and 117 (exon 4) in the KRAS gene. 

The research with the NRAS gene has revealed mutations in six cases (4.3%) in the codons 12 and 61 (exons 2 and 3), with no mutation being detected in the codon 13 (exon 2), in the codon 59 (exon 3) and in the codons 117 and 146 (exon 4). 

The immunohistochemistry slides analysis revealed marking of exclusive membrane in only four cases (2.8%) and of exclusive cytoplasm, in other four (2.8%). All cases with exclusive marking of membranes showed wild-type RAS and all cases marked with unique cytoplasmic showed mutated RAS. The rest of the cases showed positivity for both membrane and cytoplasm at variables intensifies. Twenty-three cases (16.5%) were allocated in the “positive” group (scores 2 and 3). In the “negative” group (negative scores) were allocated 49 cases (35.2%) and in the group “uncertain” (scores 0 and 1) were allocated 67 cases (48.2%, Table 3).The direct correlation of the EGFR expression stratified into classes “positive”, “negative” and “uncertain” in comparison to the mutational status of the expanded RAS showed a strong association between groups (p<0.001, Table 3). Of the 23 cases of the “positive” class, 21 (91.3%) showed the wild RAS gene for the researched mutations. Of the 49 cases of “negative” class, 41 (83.7%) presented mutation in the expanded RAS panel. The 67 cases allocated as “uncertain” presented parity in the findings with 32 cases of (47.8%) RAS wild and 35 mutated cases (52.2%).

Test validation EGFR membrane-cytoplasmic expression is shown in Table 4.

**TABLE 3:** Correlation between the EGFR groups and the mutational condition of the RAS

EGFR							
RAS	Positive		Negative		Uncertain		p *
	n	%	n	%	n	%	
Savage	21	91.3	8	16.3	32	47.8	<0.001
Mutated	2	8.7	41	83.7	35	52.2	
Total	23	100.0	49	100.0	67	100.0	

* Chi-Squared Test.

**TABLE 4:** Test validation EGFR membrane-cytoplasmic expression by immunohistochemistry considering only the positives and negatives

EGFR	RAS		
	Savage	Mutated	Total
Positive	21	2	23
Negative	8	41	49
Total	29	43	72

Sensibility=72.4%; specificity=95.3%; accuracy=86.1%; predictive positive value=83.6%; predictive negative value=86.1%

## DISCUSSION

### Relation between EGRF and RAS

Our study shows that the cytoplasmic-membrane analysis of the EGFR stratified into the classes “positive”, “negative” and “uncertain” based on the regulation of the receptor is able to predict the mutational status of the RAS in 51.7% of the analyzed cases, with 86.1% of accuracy (p<0.001, Table 4).

The EGFR activates the MAPK pathway through the RAS. The same will result in the transcription of various growth factors; however, there is also the production of MIG-6 which acts by activating the internalization and degradation of the receptor^2^. Studies investigating the direct relationship between EGFR amplification and expression and the mutation status of the KRAS, showed no relationship between these two components^8^. Nevertheless, they were performed in small series, some analyzed only the amplification^8^ and the others studied the expression by analysis of high and low affinity for binding to the receptor^19^, and that there have been no studies, like ours, comparing the EGFR cytoplasmic-membrane immunohistochemistry expression using the antibody clone (DAK-H1-WT) and the mutational status of the expanded RAS.

### Gene and EGFR receptor

EGFR is a transmembrane receptor, part of the Erb’s family. Has an extracellular domain that can be selectively activated by epidermal growth factor (EGF) and alpha growth transforming factor (TGF-α), leading to dimerization and activation of the receptor. Once activated the receptor suffers auto phosphorylation of its tyrosine-kinase intra-cytoplasmic domain there in cascade activate the RAS/RAF/MEK/MAPK and therefore the nuclear transcription factors by activation and regulation of genes responsible for cellular replication, angiogenesis, differentiation, etc. Expressed in lung, colon, breast, head and neck, ovarian cancer, pancreas, bladder and kidney tumors^12^ ^,^ ^26^. 

The expression and activation of the receptor have early and late regulatory loops, acting on signal attenuation across the cascade and/or in the internalization of the receptor. As a result, there is dephosphorization or degradation of the EGFR^2^ ^,^ ^3^ ^,^ ^5^ ^,^ ^18^, allowing its detection in both membrane and cytoplasm, justifying the cytoplasmic-membrane joint analysis in the IHC for determination of the marking score^14^.

The mutations of the EGFR gene are uncommon in colorectal carcinomas, enabling the use of the wild-type clone DAK-H1-WT for the research of the receptor. There are studies by immunohistochemistry showing EGFR expression in 60-80% of the CCR^25^; however, only 24% of these tumors presented amplification of the EGFR gene^23^. Clinical studies showed a discrepancy between the EGFR expression and the therapeutic response to the receptor inhibitors^17^, and that up to 25% of the tumors with negative expression for EGFR are responsive to Cetuximab (iEGFR)^4^. But the vast majority of these studies used the kit PharmDx^TM^ with the antibody EGFR2.5 in the immunohistochemistry reactions for detection of EGFR, contrasting with the significant increase of sensitivity in these receptors using the clone of antibody DAK-H1-WT that detects only wild-type receptors^26^. Our study showed 97.2% of EGFR membrane expression, confirming the great gain in sensitivity with this clone.

While some studies showed a relationship between the expression and the degree of the tumor or the survival of the patients^7, 20^, others did not have similar results^25^, as well as in our analysis in which no significant correlation was found between the EGFR expression in comparison to age, gender, location, histological type, histological grade and stage.

A study with 47 patients showed no significant of amplification of the gene in the therapeutic response^9^. Another showed that the 17% of the patients with wild KRAS and amplification of the EGFR gene treated with Cetuximab had therapeutic response^13^. Similar percentage to the “positive” cytoplasmic-membrane score of our study, in which 16.5% of the cases showed a strong correlation with the wild expanded RAS, suggesting that there may be a correlation between this study group and the amplification of the gene. Unlike the “uncertain” group where the cases can be amplified but not hyper-expressed due to inhibition by the regulatory loops of the mutated RAS, constitutively activated, or cases in which there is no amplification and therefore doubtful expression of the receptor. Further analysis correlating the cytoplasmic-membrane to the amplification of the EGFR gene and the mutational status of the RAS are needed to clarify these issues.

### Genes and KRAS and NRAS proteins

KRAS and NRAS are members of the family of RAS oncogenes. They are binder proteins, initiator of the MAPK pathway after activation by the EGFR and codified by specific RAS proto-oncogenes. The HRAS, third member of the RAS family, is rarely mutated in CCR^6^. Somatic mutations of these genes are early event in carcinogenesis, present in 40% to 45% of CCR, usually being mutually exclusive^15^ ^,^ ^21^. This percentage is lower than one found in our analysis (56.1%) that considered all the exons already recommended in current guidelines^5^ ^,^ ^18^. Our study also showed a mutation of the NRAS in 4.3% of the cases, value also higher than the 2% found in a previous study^18^. 

Once mutated, these proteins stimulate the EGFR/MAPK pathway constitutively, therefore leading to resistance to treatment with inhibitors of this receptor (Cetuximab and Panitumumab)^1^ ^,^ ^11^ ^,^ ^14^ ^,^ ^28^. Cases with wild KRAS presented a clinical benefit with the use of inhibitors of EGFR alone and in combination with chemotherapy^16^ ^,^ ^27^. However, only 13-17% of the tumors with wild KRAS responded^1^ ^,^ ^11^. Phase 3 randomized clinical studies showed the lack of response in patients with mutations in the KRAS or NRAS^28^. BRAF mutations (p.V600E), molecule also associated with the signaling of RAS-EGFR has been implicated in a proportion of non-responsive patients^21^ ^,^ ^27^. These mutations could justify cases of the “negative” group of our study in which the RAS mutation was not detected, since these genes are also linked to the receptor regulatory loops, which may decrease its expression.

There is also a parallel pathway of the PTEN/PIK3CA/AKT - EGFR that inhibits the apoptosis of neoplastic cells, being that the PTEN (Phosphatase and Tensin Homologue Gene) inhibits this pathway when using the PIK3CA as substrate. Mutations in the pathway lead to hyper-phosphorylation of the AKT, constitutive inhibition of apoptosis and consequent failure in the response of the EGFR inhibitors^10^.

The limitations of this study are: small sample; the analysis of the EGFR expression was performed by only two pathologists; the mutational status of the RAS was available during the expression analysis; the mutational status of the BRAF and PIK3CA genes were not contemplated in the study.

This study is pioneer in the analysis of the EGFR expression by immunohistochemistry, using a cytoplasmic-membrane marking score stratified into the classes “positive”, “negative” and “uncertain”, based on the recycling of this receptor triggered by regulation loops, it is able to predict the mutational status of the RAS in 51.7%of cases with 86.1% of accuracy. However, further studies are needed to determine the reason why almost half of the cases are still uncertain. Analyzes contemplating the EGFR amplification and mutations in other genes of the EGFR/MAPK cascade as BRAF and PIK3CA, may allow a better stratification of this population.

## CONCLUSIONS

The cytoplasmic-membrane analysis of the EGFR expression stratified into “positive”, “negative” and “uncertain” predicts mutational status of the RAS in 51.7% of the cases (p<0.001), with 86.1% of accuracy.
